# A Detailed Analysis of Clinical Features and Outcomes of Patients with Pyogenic Spondylodiscitis Presenting without Axial Back Pain

**DOI:** 10.3390/tropicalmed6020054

**Published:** 2021-04-20

**Authors:** Luigi Aurelio Nasto, Massimo Fantoni, Valerio Cipolloni, Luca Piccone, Enrico Pola, Alfredo Schiavone Panni

**Affiliations:** 1 Department of Paediatric Orthopaedics, IRCCS Istituto “G. Gaslini”, via G. Gaslini 5, 16147 Genoa, Italy; luigiaurelionasto@gaslini.org; 2 Department of Infectious Disease, “A. Gemelli” University Hospital, Università Cattolica del Sacro Cuore di Roma, l.go A. Gemelli 1, 00168 Rome, Italy; massimo.fantoni@unicatt.it; 3 Department of Trauma and Orthopaedics, “A. Gemelli” University Hospital, Università Cattolica del Sacro Cuore di Roma, l.go A. Gemelli 1, 00168 Rome, Italy; valeriocipolloni@gmail.com (V.C.); l.piccone1992@gmail.com (L.P.); 4 Department of Orthopaedics, A.O.U. “Vanvitelli” University Hospital, “Luigi Vanvitelli” University, Via del Sole 10, 80138 Naples, Italy; alfredo.schiavonepanni@unicampania.it

**Keywords:** pyogenic spondylodiscitis, spinal infections, microbiology of spinal infections, classifications of spinal infections, surgical treatment of pyogenic spondylodiscitis

## Abstract

Study design: Retrospective analysis of a single institution prospective, longitudinal database of spinal pyogenic infections. Diagnosis of pyogenic spondylodiscitis (PS) can be challenging. Although presenting symptoms are often non-specific, acute non-remitting axial back pain is the most striking feature. Nevertheless, several authors have reported on the uncommon occurrence of patients with PS without axial back pain. The aim of this study was to characterize presenting symptoms, causative agents, comorbidities, and treatment outcomes of patients presenting with painless pyogenic spondylodiscitis. A total of 214 patients diagnosed with PS were reviewed; patients were divided into two groups: patients presenting with no axial back pain (*no pain group*, n = 16), and patients presenting with axial back pain (*control group*, n = 198). Analyzed data comprised general demographics, presenting symptoms, comorbidities, spinal infection location, and amount of spinal involvement. While average age (62.4 vs. 65.0) and sex distribution was similar between the two groups, a significant diagnostic delay was noted in the control group (53 vs. 17 days, *p* < 0.001). Patients in the no pain group were more likely IV drug abusers or have had liver failure/cirrhosis. Anatomic distribution (i.e., cervical vs thoracolumbar) of the infection did not differ between the two groups, but a higher number of post-surgical infections was noted in the no pain group (37.5 vs. 15.6%, *p* = 0.026). *E. coli* and Pseudomonas spp. were more commonly seen in no pain group patients, and mortality was also higher in this group (12.5 vs. 6.0%, *p* = 0.004).

## 1. Introduction

Spinal infections represent 3 to 5% of all cases of osteomyelitis, with incidence ranging from 0.5 to 5.8/100,000 inhabitants/year [1]. The term pyogenic spondylodiscitis (PS) is used to describe a spectrum of spinal infections encompassing vertebral osteomyelitis, spondylitis, and discitis. Although PS incidence is higher in elderly and chronically debilitated patients, in recent years, an increasing incidence has been reported in young adults due to immunodeficiency syndromes, drug abuse, and invasive surgical procedures on the spine. Hematogenous spread remains the most common route of infection, with urogenital, pulmonary, infective endocarditis, or diabetic foot infections being the most common source of infection. PS may also be caused by direct inoculation of bacteria into the disc space by penetrating wounds, spine surgery, or diagnostic procedures. The inflammatory process due to infection can lead to progressive bone destruction, kyphosis, and neurological compromise in the absence of appropriate treatment [1,2,3].

Diagnosis of PS can be challenging; a significant diagnostic delay between the onset of symptoms and definitive diagnosis has been reported by several authors [4]. Presenting symptoms are non-specific and can be masqueraded by other coexisting conditions (e.g., previous spinal surgery, cardiovascular conditions, diabetes). The most common and striking finding in patients with PS is back pain [5,6]. Occasionally, pain radiates to lower or upper limbs, but in all cases, it is described as stabbing, very intense pain present at night, which worsens with weight-bearing. Only 50% of patients present with fever, while marked tenderness of the paraspinal muscles is present in 75 to 95% of patients. Neurological deficit is much less common and can be the presenting symptom in up to 20% of patients [7]. A definitive diagnosis of PS is usually confirmed by microbiological confirmation and spinal magnetic resonance imaging.

Once a diagnosis of PS is established, the mainstay of treatment remains IV antibiotic therapy. In patients without neurological compromise or major spinal instability, conservative orthopedic treatment with rigid immobilization is indicated. In case of spinal instability or impending neurological deficit, surgical decompression and debridement are advised.

In 2008, we established a prospective, longitudinal database for all patients with spinal infections treated at our institution [8]. Several clinical, radiographical, microbiological, and surgical data are regularly collected. The ultimate goal of our research endeavor is to provide a more standardized approach for the treatment of these complex patients. In a recent review of the database, we noticed a small percentage of patients presenting with no pain but a confirmed diagnosis of pyogenic spondylodiscitis. This is an uncommon clinical scenario that can determine a significant delay in diagnosis and deserves further investigation. The aim of this manuscript is to provide a detailed analysis of presenting symptoms, clinical picture, and comorbidities, along with treatment data of these patients.

## 2. Materials and Methods

Following Institutional Review Board (IRB) approval, we retrospectively reviewed all consecutive patients with a confirmed spinal infection enrolled in a longitudinal, single-center, prospective database from January 2008 to December 2018. All patients gave their written consent at the enrolment into the database. For the current analysis, only patients with a diagnosis of pyogenic spondylodiscitis and a minimum 2-year follow-up were enrolled. A diagnosis of pyogenic spondylodiscitis was made on clinical (i.e., symptoms suggestive of PS with laboratory abnormalities of increased white blood cell count, erythrocyte sedimentation rate, and C-reactive protein), radiological (i.e., abnormal MRI and CT scans), and microbiological (i.e., isolation of the causative agent and/or typical histologic pattern) grounds. All data were collected by independent researchers not involved in data analysis or clinical care of the patients.

For the current analysis, patients were divided in two groups: patients presenting without low back pain at diagnosis (*no pain group*), and patients presenting with axial low back pain (*control group*).

Demographics and clinical data were collected at the initial clinical encounter. Patients were followed at regular intervals until the complete healing of the infection. X-ray, CT, and MRI imaging were obtained in all patients, and pyogenic spondylodiscitis was classified as previously reported [8]. Antibiotic therapy was instituted and monitored by a dedicated Infectious Diseases team of our hospital according to the most recent available evidence and guidelines [9].

A healed infection was considered after a complete course of antibiotic therapy (minimum 6 weeks), negative blood inflammatory markers, absent clinical findings (i.e., pain, paraspinal muscle tenderness, fever) 12 months after the end of treatment. A relapsed infection was defined as a pyogenic spondylodiscitis presenting at the same spinal level of the previous infection within 12 months. Treatment failure was defined as persistence of symptoms (i.e., pain) and raised inflammatory markers (i.e., CRP > 5 mg/dL) at the end of antibiotic treatment [9]. Treatment outcomes were defined as: (1) *full recovery*, namely survival and complete disappearance of all signs and symptoms with no residual disability, (2) *qualified recovery*, survival, and disappearance of all sign and symptoms but with a residual disability such as motor weakness or chronic pain due to spinal imbalance, spinal instability, bone destruction, residual spinal canal stenosis, (3) *death*, either caused by the infection or by associated comorbidities [10].

### Statistical Analysis

Data are expressed as mean with ranges unless stated otherwise; counts and percentages are used as appropriate. The normality distribution of the data was assessed using the Shapiro–Wilk test. Categorical variables were compared using χ^2^ test, while continuous variables were compared using Student’s *t*-test. A *p* level ≤ 0.05 was considered significant. Data were analyzed using the SPSS Statistics software, v23 (SPSS, IBM, Armonk, NY, USA) and Microsoft Office Excel 2016 Professional (Microsoft, Redmond, WA, USA).

## 3. Results

A total of 214 patients with confirmed pyogenic spondylodiscitis (PS) were included in our institutional database from January 2008 through December 2018. Among these, 16 patients (7.5%, 8 males, no pain group) presented with no axial back pain at the time of diagnosis (*no pain group*), while 198 patients (92.5%, 130 males, control group) presented with back pain (*control group*). With the exception of back pain, presenting symptoms were not statistically different between the two groups (Table 1).

At presentation, a raised temperature was observed in 68.8 and 60.6% (*p* = 0.693), weight loss in 31.3 and 24.7% (*p* = 0.564), and radiating pain in 18.7 and 41.4% (*p* = 0.074) of the no pain and control groups, respectively. The average C-reactive protein level was also not significantly different between the two groups (95.1 mg/L vs. 69.7 mg/L in no pain and control group, respectively). The average time to diagnosis in the control group was 53 days (range 2 to 300). This was significantly longer than the diagnostic delay observed in the no pain group (17 days, range 7–30, *p* < 0.001) (Table 1).

Comorbidity distribution among the two group was also similar, with the exception of a higher percentage of IV drug abuse (18.8 vs. 4%, *p* = 0.010) and liver failure/cirrhosis (18.8 vs. 3.0%) in the no pain group. Prevalence of diabetes (12.5 vs. 28.3%, *p* = 0.171), immunodepression (18.8 vs. 7.6%, *p* = 0.121), and cancer (37.5 vs. 20.7%) was similar in the two groups. The average number of comorbidities per single patient was on average higher in the no pain group (2.5 vs. 1.8) but not statistically significant (*p* = 0.153) (Table 1).

The level of the infection was more commonly located in the lumbar spine in both groups (68.7 vs. 69.7%, *p* = 0.936), followed by thoracic spine (37.5 vs. 31.3%, *p* = 0.609). In 56.2% (no pain group) and 73.2% (control group) of cases a single level infection was observed (*p* = 0.145). The number of multiple level infection and the extent of spinal involvement (i.e., type A, B and C infection) was not statistically different between the two groups (Table 2). Interestingly, post-surgical infection was more common in the no pain group (37.5 vs. 15.6%, *p* = 0.026).

Microbiology isolates for the two groups are shown in Table 3.

*S. aureus* was the most common causative agent in both groups, with a similar prevalence between the two groups (27.2 vs. 35.2%). A higher number of *E. coli* (22.2 vs. 5.8%, *p* = 0.005) and *Pseudomonas* (11.1 vs. 1.4%, *p* = 0.005) infections was observed in the no pain group.

While antibiotic therapy was instituted in all patients, 25 and 23.2% of patients in the no pain and control group, respectively, required surgical intervention. The most common operation performed was a decompression and debridement (50 vs. 63%), while a posterior stabilization was required in 50 and 30.4% of patients in the no pain and control groups, respectively (*p* = 0.322) [11]. The average number of days of admission was 59.3 in the no pain group and 51 in the control group (*p* = 0.391). The observed mortality was 12.5% in the no pain group and 6% in the control group, and this was statistically significant (*p* = 0.004) (Table 4).

## 4. Discussion

**Figure 1:**
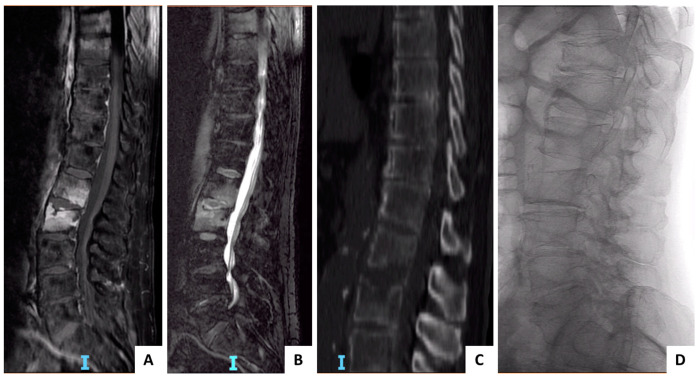
(**A**–**D**) Case example of L1–L2 pyogenic spondylodiscitis presenting without low back pain. An 86 years-old female patient with a history of chronic obstructive pulmonary disease (COPD), cigarette smoking, Alzheimer’s disease, and recent history of repeated bladder catheterization due to urinary retention. At presentation, the patient had been complaining of low-grade fever for the last 3 weeks and no low back pain. Blood tests show increased C-reactive protein (25 mg/dL) and white blood cell counts (12.4 × 10^3^/mm^3^). A chest X-ray followed by a chest CT scan were ordered at first but showed no signs of infection. Repeated blood tests showed raised gamma fraction in serum protidogram, but a whole body skeletal X-ray did not show any sign of osteolytic lesions. A whole spine MRI exam was ordered with a query diagnosis of multiple myeloma. MRI exam (panels A and B) showed the presence of an L1–L2 spondylodiscitis, which was further confirmed by CT scan (panel C). The patient underwent CT guided biopsy and *E. coli* was identified as the causative agent. IV antibiotic therapy with ceftriaxone was started for 3 weeks and followed by 3 more weeks of oral amoxicillin/clavulanic acid. Complete healing of the infection was achieved with no residual biomechanical instability on standing X-rays examination at the end of treatment (panel D).

Even today, with modern medicine and novel antibiotic therapies available, pyogenic spondylodiscitis remains a difficult clinical diagnosis and a severe disease to the patient. Presenting symptoms are frequently non-specific [12]. Back pain, fever, weight loss, radiating pain to the upper or lower limbs, or more rarely, neurological deficits, have been reported as common presenting symptoms by many authors. Nevertheless, many other clinical conditions can mimic the presenting symptoms of PS (e.g., cancer), and often, diagnosis is delayed (Figure 1) [13]. Back pain is perhaps the only clinical symptom that can help the clinician to localize the patient’s problem to the spine. In this manuscript though, we present a group of patients with a confirmed PS with no back pain.

Several authors have previously reported on patients with PS presenting without back pain. The percentage of no pain patients varies in the literature from 2.5 to 15%, and it was 7.5% in our case series [1,2,14]. The mechanisms by which patients present without back pain are not entirely known. While inner parts (i.e., nucleus pulposus and inner annulus fibrosus) of the disc are not innervated, nociceptors have been described in the outer part of the annulus fibrosus and vertebral endplates. Progressive disc degeneration has been associated with vascular ingrowth accompanied by nerve fibers growing in the inner parts of the intervertebral disc [15]. Therefore, one could speculate that infections localized in the inner parts of the disc might not present with pain, while infections localized in the outer parts of the disc or in the proximity of nerve structures might account for severe axial back pain. In our study, spinal involvement by the infection was classified according to Pola et al. in type A, B, and C [8]. This classification provides a simple and reliable way to gauge the extent of involvement of the spinal motion by the infection, including segmental instability and neurological compromise. As shown in Table 2, no significant difference was noted in type distribution among the two groups. Another hypothesis is that patients without axial back pain might present with a more localized infection and a less pronounced inflammatory reaction [2]. However, our data show that patients with no pain have similar levels of CRP (*p* = 0.348) and WBC (*p* = 0.289) compared to pain-presenting patients. Furthermore, prevalence of diabetes among the two groups in our case series was also similar (12.5 vs. 28.3%, *p* = 0.171). Hence, we were not able to identify any specific factor related to no pain presentation in patients with PS. Therefore, maintaining a high grade of suspicion remains the most important aspect in the early identification of patients with pyogenic spondylodiscitis.

Interestingly, no pain presentation was associated with a reduced diagnostic delay in our patients (17 days vs. 53 days, *p* < 0.001). A significant diagnostic delay in the diagnosis of PS has been reported by several authors [2,6,7]. Reasons for the delay are varied, but mostly due to the non-specific presentation of the symptoms or the presence of concomitant infections. Our findings of reduced diagnostic delay in patients presenting without low back pain may seem counterintuitive at first. While we do not have a definitive explanation for this, one must notice that all these patients were brought to our attention because of symptoms other than low back pain. This stimulated further workup in all patients and ended with the identification of a suspect spinal infection (Figure 1). On the other hand, in patients complaining of low back pain, the symptom was overlooked at first because it is non-specific and widely prevalent in the adult population and this might explain the observed diagnostic delay.

In our case series, the prevalence of IV drug abusers was significantly higher in the no pain group (18.8 vs. 4.0%, *p* = 0.010). Wang et al. have specifically looked at clinical features of patients with PS and IV drug abuse. In their report, 40% of IV drug abusers presented without pain but with neurological involvement in 61% of cases [16]. In our case, a series neurological involvement was present in 18.8% and was not significantly different from the control group. Taken together, these findings suggest that IV drug abusers are at higher risk for no pain presentation compared with other groups of patients.

Interestingly, no pain presentation was also more common in post-operative PS in our case series. An interesting study on post-operative PS patients was published by Dufour et al., where the authors compared 7 patients with post-operative PS vs. 16 patients with spontaneous PS. The authors noted a longer delay in diagnosis in post-operative PS as well as a higher incidence of neurological deficit [17]. However, while Dufour et al. reported a 100% prevalence of back pain in post-operative PS patients, we did observe six patients with post-operative spondylodiscitis in the no pain group presenting without axial back pain.

Arguably, the most important finding in our study was the higher mortality rate observed in PS patients presenting without back pain (12.5 vs. 6.0%, *p* = 0.004). The overall mortality rate for PS is 5–10% [18]. The higher observed mortality rate in no pain patients can be partially explained by the higher prevalence of iv drug abusers and liver cirrhosis among our patients. 

There are some limitations to this study. Although all data were entered prospectively in a longitudinal database, this was a retrospective analysis. Furthermore, the limited number of patients in the no pain group (16 vs. 198) does not allow us to draw definitive conclusions about this varied population of patients.

## 5. Conclusions

In conclusion, our study confirms previous reports that back pain can be absent in up to 7.5% of patients with PS. Patients presenting without pain are more commonly IV drug abusers or have liver cirrhosis. The average number of comorbidities per patient tends to be higher in patients without back pain. A higher number of post-operative infections were observed in these patients with a higher number of *E. coli* and *Pseudomonas* isolates. Although the percentage of patients requiring surgery was similar between no pain and control PS group, mortality from PS was significantly higher in patients without back pain (12.5 vs. 6.0%).

## Figures and Tables

**Table 1:** Baseline demographics at diagnosis.

	No Pain Group (*n* = 16)	Control Group (*n* = 198)	*p*
Age (yrs)	62.4 (35–86)	65.0 (25–88)	0.501
Sex (M:F)	8 M:8 F	130 M:68 F	0.208
Diagnostic delay (days)	17.0 (7–30)	53.0 (2–300)	**<0.001**
Presenting symptoms			
Axial back pain (%)	0 (0.0)	198 (100.0)	**<0.001**
Radiating pain (UL - LL)	3 (18.7)	82 (41.4)	0.074
Fever (%)	11 (68.8)	120 (60.6)	0.693
Weight loss (%)	5 (31.3)	49 (24.7)	0.564
Neurological impairment (%)	3 (18.8)	46 (23.2)	0.681
Spinal cord compromise (%)	0 (0.0)	36 (18.2)	0.053
Raised WBC count (>10,000/mm^3^)	12.9 (8.4–17.4)	12.4 (9.6–15.1)	0.289
C Reactive Protein (mg/dL)	95.1 (4.9–298.3)	69.7 (4.9–397.0)	0.348
Comorbidities			
Cigarette smoking (%)	5 (31.3)	50 (25.3)	0.597
Iv drug abuse (%)	3 (18.8)	8 (4.0)	**0.010**
Long term steroid therapy (%)	1 (6.2)	26 (13.1)	0.425
Alcohol consumption (%)	0 (0.0)	4 (2.0)	0.566
Immunodepression (%)	3 (18.8)	15 (7.6)	0.121
Diabetes (%)	2 (12.5)	56 (28.3)	0.171
Cancer (%)	6 (37.5)	41 (20.7)	0.118
CVD (%)	6 (37.5)	67 (33.8)	0.766
Liver cirrhosis (%)	3 (18.8)	6 (3.0)	**0.002**
Chronic renal failure (%)	3 (18.8)	24 (12.1)	0.442
Other (%)	2 (12.5)	26 (13.1)	0.942
Comorbidities (range)	2.5 (0–5)	1.8 (0–8)	0.153

Data are expressed as mean (range) unless stated otherwise. M, males; F, females; UL, upper limbs; LL, lower limbs; WBC, white blood cells; IV, intravenous; CVD, cardiovascular disease.

**Table 2:** Location and classification (*n*, %).

	No Pain Group (*n* = 16)	Control Group (*n* = 198)	*p*
Lumbar Spine	11 (68.7)	138 (69.7)	0.936
Type A	7 (64)	72 (53)	
Type B	3 (27)	24 (17)	
Type C	1 (9)	42 (30)	
Thoracic Spine	6 (37.5)	62 (31.3)	0.609
Type A	1 (17)	15 (24)	
Type B	3 (50)	26 (42)	
Type C	2 (33)	21 (34)	
Cervical Spine	0 (0.0)	18 (9.0)	0.207
Type A	0 (0)	2 (11)	
Type B	0 (0)	2 (11)	
Type C	0 (0)	14 (78)	
Single level	9 (56.2)	145 (73.2)	0.145
Multiple levels Contiguous Multiple skip lesions	7 (43.8) 5 (71) 2 (29)	53 (26.8) 32 (60) 21 (40)	
Haematogenous spread	10 (62.5)	107 (54.0)	0.513
Post-surgical	6 (37.5)	31 (15.6)	**0.026**

Data are expressed as the number of isolates (percentage) unless stated otherwise. Classification of types A, B, and C refers to Pola et al. [8]. Type A, no instability, no neurological deficit, no epidural abscess; Type B, significant bone destruction and instability, no neurological deficit, no epidural abscess; Type C, epidural abscess or neurological deficit regardless of bone destruction/instability.

**Table 3:** Microbiology isolates for the two groups.

	No Pain Group (*n* = 16)	Control Group (*n* = 198)	*p*
*S. aureus*			
MSSA	4 (22.2)	60 (28.9)	0.655
MRSA	1 (5.5)	13 (6.3)	0.960
Coagulase negative S.	1 (5.5)	34 (16.4)	0.255
*Streptococcus*	0 (0.0)	27 (13.0)	0.103
*Enterococcus*	2 (11.1)	7 (3.4)	0.085
*E. coli*	4 (22.2)	12 (5.8)	**0.005**
*Proteus*	0 (0.0)	4 (1.9)	0.566
*Klebsiella*	0 (0.0)	7 (3.4)	0.444
*Pseudomonas*	2 (11.1)	3 (1.4)	**0.005**
*Bacteroides*	0 (0.0)	2 (0.9)	0.686
Others	4 (22.2)	36 (17.4)	0.571
Yeasts	0 (0.0)	2 (0.9)	0.686
Polimicrobials	2 (11.1)	9 (4.3)	0.165

Data are expressed as the number of isolates (percentage) unless stated otherwise.

**Table 4:** Treatment provided.

	No Pain Group (*n* = 16)	Control Group (*n* = 198)	*p*
Surgical intervention	4 (25.0)	46 (23.2)	0.872
Decompression & Debridement	2	29	
Posterior stabilization	2	17	
Anterior debridement	0	0	
Combined approach	0	0	
Days of admission	59.3 (14–130)	51 (5–300)	0.391
Full recovery	10 (62.5)	134 (67.7)	0.170
Qualified recovery	4 (25.0)	40 (20.2)	0.647
Relapse	1 (6.2)	11 (5.5)	0.907
Mortality	2 (12.5)	12 (6.0)	**0.004**

Data are expressed as the number of patients (percentage) unless stated otherwise.

## Data Availability

Data available on request due to privacy restrictions.
